# Effect of ghost pepper on cell proliferation, apoptosis, senescence and global proteomic profile in human renal adenocarcinoma cells

**DOI:** 10.1371/journal.pone.0206183

**Published:** 2018-10-31

**Authors:** Venu Perla, Marjan Nadimi, Rishi Reddy, Gerald R. Hankins, Padma Nimmakayala, Robert T. Harris, Jagan Valluri, Cristian Sirbu, Umesh K. Reddy

**Affiliations:** 1 Gus R. Douglass Land-Grant Institute and Department of Biology, West Virginia State University, Institute, West Virginia, United States of America; 2 Department of Biological Sciences, One John Marshall Drive, Marshall University, Huntington, West Virginia, United States of America; 3 Center for Cancer Research, Charleston Area Medical Center, SE, Charleston, West Virginia, United States of America; University of South Alabama Mitchell Cancer Institute, UNITED STATES

## Abstract

Chili peppers are an important constituent of many foods and contain medicinally valuable compounds, such as capsaicin and dihydrocapsaicin. As various dietary botanicals have anticancer properties, this study was aimed to examine the effect of Ghost pepper (Bhut Jolokia), one of the hottest chili peppers in the world, on cell proliferation, apoptosis, senescence and the global proteomic profile in human renal cell adenocarcinoma in vitro. 769-P human renal adenocarcinoma cells were cultured on RPMI-1640 media supplemented with fetal bovine serum (10%) and antibiotic-antimycotic solution (1%). Treatment stock solutions were prepared in ethanol. Cell proliferation was tested with phenol red-free media with capsaicin (0–400 μM), dihydrocapsaicin (0–400 μM), capsaicin + dihydrocapsaicin (5:1), and dry Ghost peppers (0–3 g L^-1^) for 24, 48 and 72 h. Polycaspase and senescence associated-beta-galactosidase (SA-beta-gal) activities were tested with capsaicin (400 μM), dihydrocapsaicin (400 μM), capsaicin (400 μM) + dihydrocapsaicin (80 μM), and ghost pepper (3 g L^-1^) treatments. Global proteomic profile of cells in control and ghost pepper treatment (3 g L^-1^) was analyzed after 6 h by a shotgun proteomic approach using tandem mass spectrometry.

At 24 h after treatment (24 HAT), relative to control, cell proportion with capsaicin (400 μM), dihydrocapsaicin (400 μM), capsaicin (400 μM) + dihydrocapsaicin (80 μM), and ghost pepper (3 g L^-1^) treatments was reduced to 36%, 18%, 33% and 20%, respectively, and further reduced at 48 and 72 HAT. All treatments triggered an early polycaspase response. SA-beta-gal activity was normal or suppressed with all treatments. About 68,220 protein isoforms were identified by shotgun proteomic approach. Among these, about 8.2% were significantly affected by ghost pepper. Ghost pepper regulated various proteins involved in intrinsic and extrinsic apoptotic pathways, Ras, Rb/E2F, p53, TGF-beta, WNT-beta catenin, and calcium induced cell death pathways. Ghost pepper also induced changes in proteins related to methylation, acetylation, genome stability, cell cycle check points, carbohydrate, protein and other metabolism and cellular mechanisms.

Ghost pepper exhibited antiproliferation activity by inducing apoptosis through a complex network of proteins in human renal cell adenocarcinoma in vitro.

## Introduction

**Fig 1:**
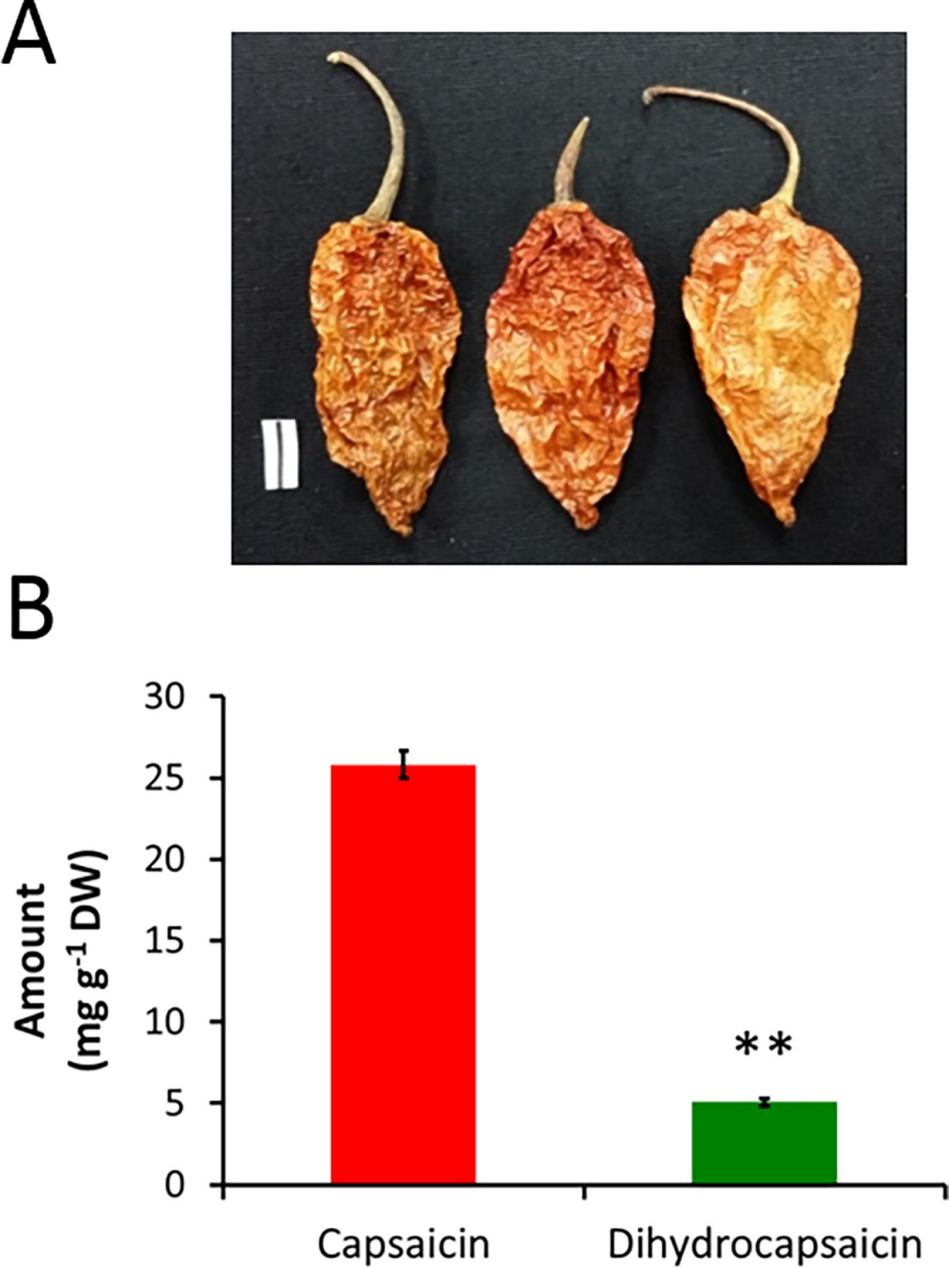
Ghost pepper and its major capsaicinoids. **(A)** Commercially available dried ripened Ghost pepper fruits. Bar = 1 cm. **(B)** Capsaicin and dihydrocapsaicin levels in the commercial dried ripened Ghost pepper powder. Values are means ± SD; n = 3; **p ≤ 0.01 (as compared to capsaicin).

‘Ghost pepper’ (also called ‘Naga chilli’ or ‘Bhoot Jolokia’) (*Capsicum chinense*, Jacq.) is one of the hottest chili peppers in the world [1] (Fig 1A). It was officially recognized by the Guinness Book of World Records as the world's hottest chili, with > 1,000,000 Scoville heat units (SHUs), in 2006 [2]. In general, chili species and varieties contain about 1% capsaicin, but the content of capsaicin in ghost pepper ranges from 2% to 4% [3,4]. These higher capsaicin levels can reduce the cost of capsaicin extraction from this chili.

Capsaicinoids are responsible for the hot or burning sensation of chili [5]. About 80% to 90% of capsaicinoids in chili fruit is capsaicin and dihydrocapsaicin [6]. Pharmacological capsaicinoids are used for pain therapy, body temperature regulation, anti-obesity treatments, and anticancer, antioxidation, and antimicrobial therapy [1].

Cancer is the second leading cause of death in the United States. About 30% to 40% of cancers could be prevented by modifying diet, maintaining optimal body weight, and regular physical activity. About 20% of cancer-related deaths annually could be prevented by increasing the consumption of vegetables and fruit. Because of their safety, low toxicity, antioxidant properties, and general acceptance as dietary supplements, fruits and vegetables are being investigated for the prevention of cancer [7]. According to an estimate based on 2009–2011 data by the US National Cancer Institute, approximately 1.6% of men and women will have a diagnosis of kidney and renal pelvis cancer at some point during their lives. In 2011, an estimated 358,603 people in the United States were living with kidney and renal pelvis cancer. Estimated new cases and deaths due to kidney cancer in 2014 in the United States were 63,920 and 13,860, respectively [8].

Animal studies reveal that ingested capsaicin is rapidly absorbed from the stomach and small intestine in animals. Subcutaneous injection of capsaicin in rats increased the blood concentration and peak concentration was reached at about 5h. The highest capsaicin levels were noticed in the kidney tissues and the lowest in the liver [9,10]. In this context, dietary consumption of chili may be a natural choice for preventing kidney cancers among men and women.

During irreversible cell death, mitotic cells can permanently arrest the cell cycle (cellular senescence) or trigger cell death programs. Among these programs, apoptosis (self-killing) and autophagy (self-eating) are well known for cell death [11]. Growing evidence supports the role of apoptosis in capsaicin-mediated responses in various cancer cell lines [1,12]. However, role of capsaicinoids in cancer cell senescence is not clear. Furthermore, investigations on a few proteins in cancer cells have led to biased and incomplete conclusions. In this regard, the objective of this study was to understand the effect of ghost pepper on cell proliferation, apoptosis, senescence and the global proteomic profile in human renal cell adenocarcinoma in vitro.

## Material and methods

### Determination of capsaicin and dihydrocapsaicin in Ghost pepper by HPLC

Ghost pepper powder was obtained from Alamo City Pepper Products, San Antonio, TX. Capsaicin and dihydrocapsaicin in the commercial Ghost pepper powder was estimated by HPLC (Waters, Milford, MA) for making equimolor concentrations [13].

### Cell culture

769-P human renal adenocarcinoma cells (CRL-1933; ATCC, Manassas, VA) were cultured in T75 or T25 flasks (Greiner, Monroe, NC) on RPMI-1640 media supplemented with 10% fetal bovine serum (FBS; ATCC) and 1% antibiotic-antimycotic solution (Gibco, Grand Island, NY) in a CO_2_ incubator at 37°C, 90% humidity, 5% CO_2_ and 21% O_2_. Cells at about 90% confluence were split (1:4 to 1:12) with 0.25% Trypsin/0.53 mM EDTA in Hank’s balanced salt solution (HBSS) without Ca^2+^ and Mg^2+^ (ATCC) for 10 min at 37 ^o^C.

### Treatments

Stock solutions of 50 mM capsaicin and 50 mM dihydrocapsaicin (Sigma-Aldrich, St Louis, MO) were prepared in ethanol, filter-sterilized and stored at -20°C. Ghost pepper powder was mixed with ethanol (30% w/v), aqua-sonicated for 2 h in ice cold water, filter-sterilized, and used as a stock solution. RPMI-1640 media without phenol red (ATCC) was supplemented with FBS (10%), L-glutamine (0.3 g L^-1^; Gibco) and antibiotic–antimycotic solution (1%) to prepare stock media for various assays. Equimolor concentrations of capsaicin (0–400 μM), dihydrocapsaicin (0–400μM), capsaicin (0–400 μM) + dihydrocapsaicin (0–80 μM) (5:1), and Ghost pepper (0–3 g L^-1^) were estimated using HPLC and prepared with stock media before various assays. Corresponding controls were prepared by mixing similar volumes of ethanol (0–0.96%) with stock media.

### Cell proliferation assay

Cell proliferation was measured with the CyQUANT cell proliferation assay kit by using a green fluorescent dye, CyQUANT GR dye, which exhibits strong fluorescence enhancement when binding to cellular nucleic acids (Life Technologies, Grand Island, NY). A total of 500 live cells were added to each well containing 200μL culture media in a 96-well flat black-bottomed plate (Greiner, Monroe, NC). After 24H (hours after treatment), culture media was replaced with freshly prepared control or treatment media. At 24, 48 and 72H after treatment, media was gently removed, and plates were kept at -80°C until further analysis. Before assay, plates were thawed for 0.5H at room temperature. In total, 200μL CyQuant GR solution (1x) was added in each well, mixed well with a multichannel pipette, and fluorescence was measured at 480/520 nm (ex/em) in a microplate reader (Synergy HT, BioTek Instruments, Winooski, VT).

### Polycaspase assay

Apoptosis was detected with the FAM FLICA Polycaspase assay kit (ImmunoChemistry Technologies, Bloomington, MN) with the green fluorescent inhibitor probe FAM-VAD-FMK that labels active caspase enzymes in living cells. Cells were cultured on phenol red-free media in T25 flasks. Cells at about 90% confluence were tested with various treatments. Staurosporine (6μM) (ImmunoChemistry Technologies) was used as a positive control. At 0.5, 1, 2 and 4H, floating cells with media were collected in a 15-mL disposable centrifuge tube and centrifuged at 5000g for 5 min at room temperature. After discarding the supernatant, cells were mixed with 600μL of 1x apoptosis wash buffer. Remaining adhered cells on the flask were lifted with trypsin, centrifuged, and mixed with the previously collected cell suspension after discarding the supernatant. FAM-FLICA poly caspase inhibitor reagent (1x) was mixed with 500μL cell suspension and incubated at 37°C for 1 h with intermittent shaking. During this incubation period, a portion of the remaining cells was used for counting the cells with a hemacytometer with trypan blue (0.04%). After incubation, 2mL wash buffer was added to cells, centrifuged, and supernatant was discarded. Again 2mL of wash buffer was added to cells and incubated at 37°C for 10min to remove excess FAM-FLICA reagent. Cells were centrifuged and the supernatant was discarded. Finally, cells were suspended in 500μL wash buffer and kept on ice. In total, 100μL cell suspension was used for estimating poly-caspase activity in 96-well flat black-bottom plates. Fluorescence was measured in the microplate reader at 488/520nm (ex/em), and the measured RFU values were normalized to total number of cells.

### SA-beta-gal assay

SA-beta-gal activity was measured with the 96-well cellular senescence assay kit (Cell Biolabs, San Diego, CA). With few exceptions, cell culture and treatment procedures were similar to polycaspase assay procedures. SA-beta-gal activity was measured at 0.5, 1 and 2H. Floating and adherent cells collected after treatment were washed with phosphate buffered saline (PBS), pooled, suspended in 400 μL cell lysis buffer (1x), and kept on ice for 10min. Then, cell suspension was dissolved by vortex. From this, 100 μL solution was frozen at -80°C for about 1 h and used it for CyQuant cell proliferation assay. From the remaining solution, 200μL was mixed with an equal amount of 2x reaction buffer containing SA-beta-gal substrate and incubated at 37°C for 1 h in the dark. After incubation, the solution was mixed by vortex, and a 200-μL solution was mixed with 800μL stop solution. From this, a 200-μL solution was used to determine SA-beta-gal activity in 96-well flat black-bottom plates. Fluorescence was measured in the microplate reader at 360/465 nm (ex/em), and RFU values were normalized to those obtained from CyQuant cell proliferation assay.

### Shotgun proteomics

Global proteomic profile for control and Ghost pepper (3 g L^-1^) treated cells at 6H were analyzed by a shotgun proteomic approach with tandem mass spectrometry. AT 6H, all the cells including floating cells were washed three times with PBS, flash frozen in liquid nitrogen and sent to BioProximity, LLC, Chantilly, VA on dry ice for shotgun proteomic analysis. Details of the proteomic analysis are given below:

### Protein denaturation and digestion

Samples were prepared for digestion with the filter-assisted sample preparation method [14]. Briefly, samples were suspended in 8M urea, 50 mM Tris-HCl, pH 7.6, 3 mM DTT, sonicated briefly, and incubated in a Thermo-Mixer at 40 ^o^C, 1000 RPM for 20 min. Samples were centrifuged and the supernatant was transferred to a 30-kD MWCO device (Millipore) and centrifuged at 13,000 g for 30 min. The buffer for the remaining sample was exchanged with 8M urea, 100 mM Tris-HCl, pH 7.6, then alkylated with 15mM iodoacetamide. The urea concentration was reduced to 2 M. Samples were digested overnight with trypsin at a ratio of enzyme to substrate of 1:100 at 37°C in a Thermo-Mixer at 1000 RPM. Digested peptides were collected by centrifugation.

### Peptide desalting

A portion of the digested peptides, about 20μg, was desalted with use of C18 stop-and-go extraction (STAGE) tips [15]. Briefly, for each sample, a C18 STAGE tip was activated with methanol, and then conditioned with 60% acetonitrile and 0.5% acetic acid, followed by 2% acetonitrile and 0.5% acetic acid. Samples were loaded onto the tips and desalted with 0.5% acetic acid. Peptides were eluted with 60% acetonitrile, 0.5% acetic acid and lyophilized in a SpeedVac (Thermo Savant) to near dryness, approximately 2h.

### Liquid chromatography-tandem mass spectrometry

Each digestion mixture was analyzed by ultra-HPLC-MS/MS. LC involved the Easy-nLC 1000 UHPLC system (Thermo). The mobile phase A was 97.5% MilliQ water, 2% acetonitrile, and 0.5% acetic acid and mobile phase B was 99.5% acetonitrile and 0.5% acetic acid. The 240-min LC gradient ran from 0% to 35% B over 210 min, then to 80% B for the remaining 30 min. Samples were loaded directly onto the column. The column was 50 cm x 75 μm ID and packed with 2 micron C18 media (Thermo Easy Spray PepMap). The LC was interfaced to a quadrupole-Orbitrap mass spectrometer (Q-Exactive, Thermo Fisher) via nano-electrospray ionization with a source via an integrated column heater (Thermo Easy Spray source). The column was heated to 50°C. An electrospray voltage of 2.2 kV was applied. The mass spectrometer was programmed to acquire, by data-dependent acquisition, tandem mass spectra from the top 20 ions in the full scan from 400–1200 m/z. Dynamic exclusion was set at 15s, singly-charged ions were excluded, isolation width was 1.6 Da, full MS resolution was 70,000 and MS/MS resolution 17,500. Normalized collision energy was set to 25, automatic gain control to 2e5, max fill MS to 20 MS, max fill MS/MS to 60 MS and underfill ratio to 0.1%.

### Data processing and library searching

Mass spectrometer RAW data files were converted to MGF format by use of msconvert [16]. Detailed search parameters are printed in the search output XML files. Briefly, all searches required 10ppm precursor mass tolerance, 0.02 Da fragment mass tolerance, strict tryptic cleavage, 0 or 1 missed cleavages, fixed modification of cysteine alkylation, variable modification of methionine oxidation and expectation value scores ≤ 0.01. MGF files in the human sequence library were searched with use of X!!Tandem [17] with both the native [18] and k-score [19] scoring algorithms and by OMSSA [20]. All searches were performed with Amazon Web Services-based cluster computed instances with the Proteome Cluster interface. XML output files were parsed and non-redundant protein sets were determined by use of Proteome Cluster [21]. MS1-based peak areas were calculated by use of XCMS [22]. Proteins were required to have ≥ 1 unique peptides across the analyzed samples with e-scores ≤ 0.001.

### Statistical analysis

Unless otherwise mentioned, all the experiments were conducted with 4 replicates, and data are presented as mean ± SD. Statistical analysis was performed using SAS software (University Edition, Cary, NC) using BASE SAS, PROC IMPORT, PROC SORT, PROC TRANSPOSE, PROC GLM, PROC PRINT and SAS MACRO programs. Significant differences among means were determined using one-way analysis of variance (ANOVA) followed by Tukey’s honestly significant differences multiple-rank test at the *p* ≤ 0.05 significance level. Proteomic data was analyzed in Microsoft Excel software using paired Student’s t-test with two-tailed distribution.

## Results

### Ghost pepper powder contained a 5:1 ratio of capsaicin to dihydrocapsaicin

HPLC analysis revealed that commercially available ghost pepper powder contained 25.80 and 5.07 mg g^-1^ dry weight (DW) capsaicin and dihydrocapsaicin, respectively. Furthermore, the ratio of capsaicin to dihydrocapsaicin was 5:1 in this Ghost pepper powder (Fig 1B).

### Adenocarcinoma cell proliferation was affected by concentration and duration of treatment

**Fig 2:**
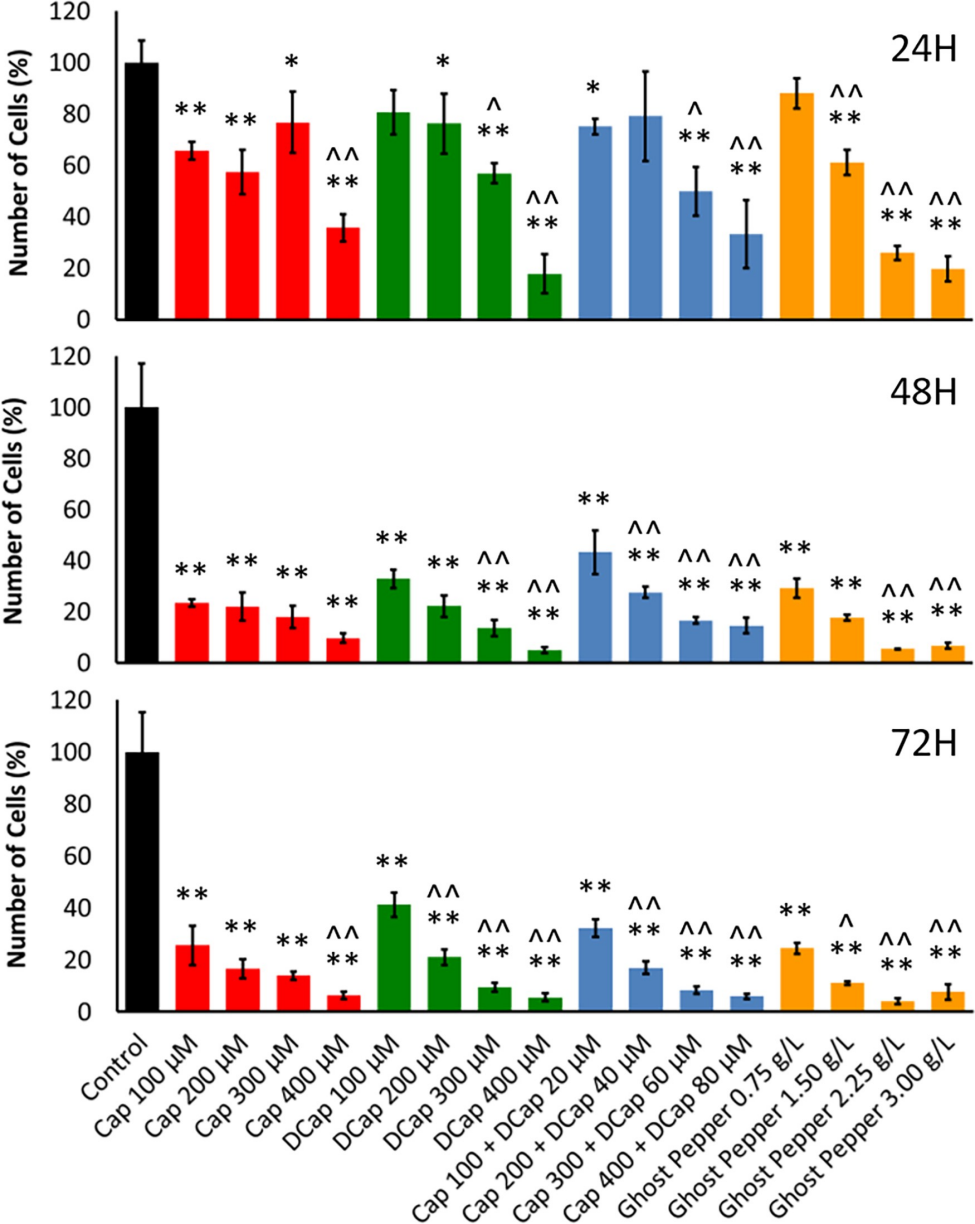
Effect of ghost pepper on human renal adenocarcinoma cell proliferation. Percentage of cells at 24, 48 and 72 H with different concentrations of capsaicin (Cap), dihydrocapsaicin (DCap), Cap + DCap (5:1), and ghost pepper. Control (media without any treatment compound) is adjusted to 100%. Values are means ± SD; n = 4; *p ≤ 0.05 and **p ≤ 0.01 (as compared to control); ^*p* ≤ 0.05 and ^^*p* ≤ 0.01 (as compared to lowest concentration with in each compound group).

**Fig 3:**
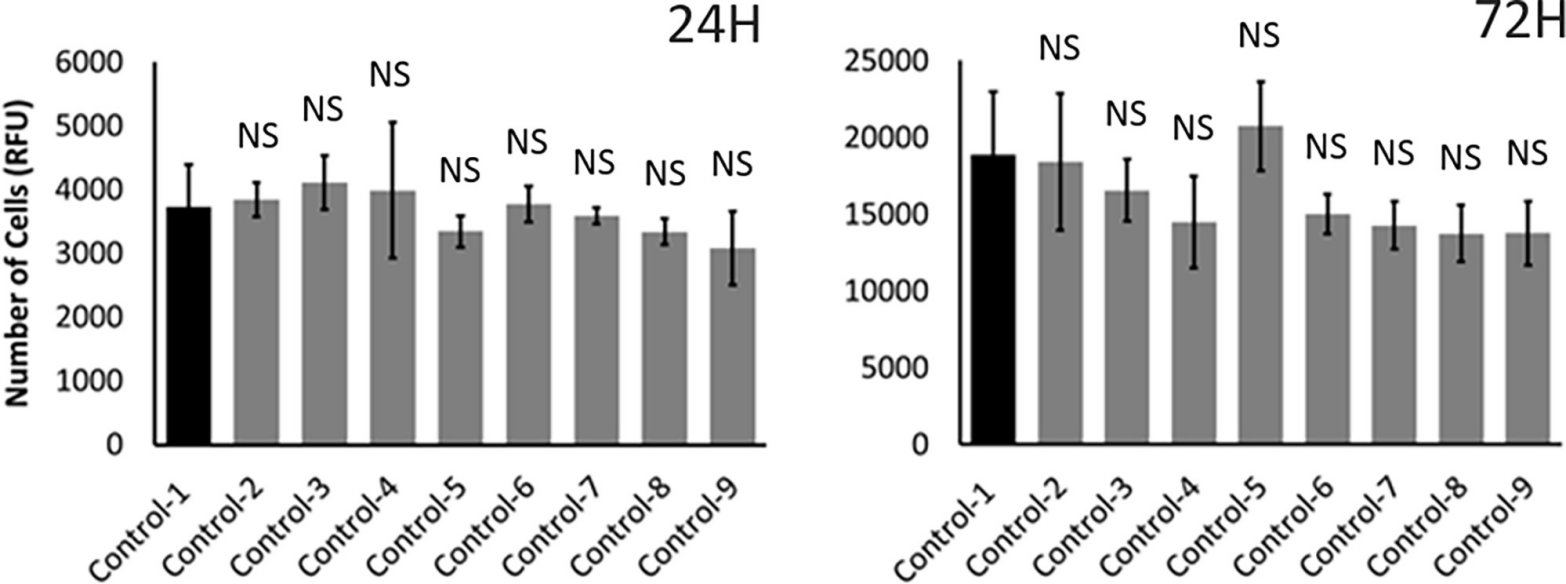
Effect of solvent on human renal adenocarcinoma cell proliferation. Different controls with different levels of ethanol, the solvent used for dissolving capsaicinoids in the study, were tested at 24 and 72 H. Control-1 contains culture media without ethanol. All other controls contain culture media with different levels of ethanol. Control-2, -3, -4 and -5 contained 0.2, 0.4, 0.6 and 0.8% ethanol, respectively. These were the corresponding controls for 100, 200, 300 and 400 μM capsaicin as well as dihydrocapsaicin treatments; and 0.75, 1.50, 2.25 and 3.00 g L^-1^ ghost pepper treatments, respectively. Control-6, -7, -8 and -9 contained 0.24, 0.48, 0.72 and 0.96% ethanol, respectively. These were the corresponding controls for 100 + 20; 200 + 40; 300 + 60; and 400 + 80 μM capsaicin + dihydrocapsaicin treatments, respectively (refer Fig 2 for treatments). Values are mean ± SD; n = 4; NS = Not significantly different from control-1 (*p* ≤ 0.05).

The proportion of adenocarcinoma cells decreased with increasing concentration of Ghost pepper and capsaicinoid treatments at 24, 48 and 72H (Hours After Treatment) (Fig 2). At 24H (Hours After Treatment), capsaicin (400μM), dihydrocapsaicin (400μM), capsaicin (400μM) + dihydrocapsaicin (80μM), and ghost pepper (3 g L-1) decreased the proportion to 36%, 18%, 33% and 20%, respectively, as compared with controls. At 48 and 72H, these values were further reduced to 5% to 15% and 6% to 8%, respectively. Various controls corresponding to different treatment combinations were tested for cell proliferation activity at 24 and 72H (Fig 3). Controls with culture media and different levels of ethanol (control-2 to control-9) did not affect cell proliferation as compared with culture media alone (control-1).

### Apoptosis versus senescence

**Fig 4:**
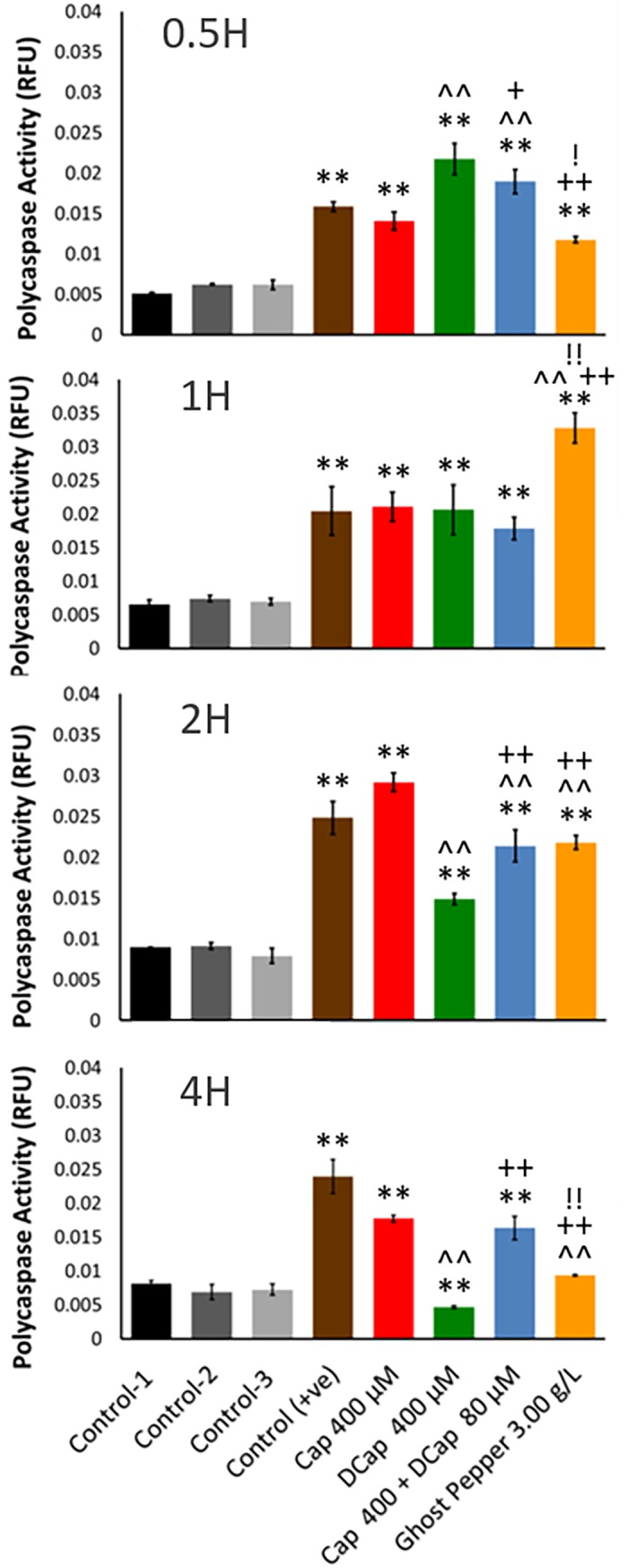
Cellular polycaspase activity in human renal adenocarcinoma cells at 0.5, 1, 2 and 4 H. Control-1 contained culture media without ethanol. Control-2 and control-3 contained culture media with different levels of ethanol (0.80 and 0.96%, respectively). Control (+ve) contained staurosporine (6 μM). Control-2 was a corresponding control for capsaicin (Cap 400 μM), dihydrocapsaicin (DCap 400 μM), and Ghost pepper (3 g L^-1^) treatments. Control-3 was a corresponding control for capsaicin + dihydrocapsaicin treatment (Cap 400 μM + DCap 80 μM) (5:1). Polycaspase activities were normalized with respective total number of cells. Values are means ± SD; n = 4; *p* ≤ 0.05 (*, ^, + or ! ); *p* ≤ 0.01 (**, ^^, ++ or !!); * or ** as compared to control-1; ^ or ^^ as compared to cap 400 μM; + or ++ as compared to DCap 400 μM; ! or !! as compared to Cap 400 μM + DCap 80 μM.

**Fig 5:**
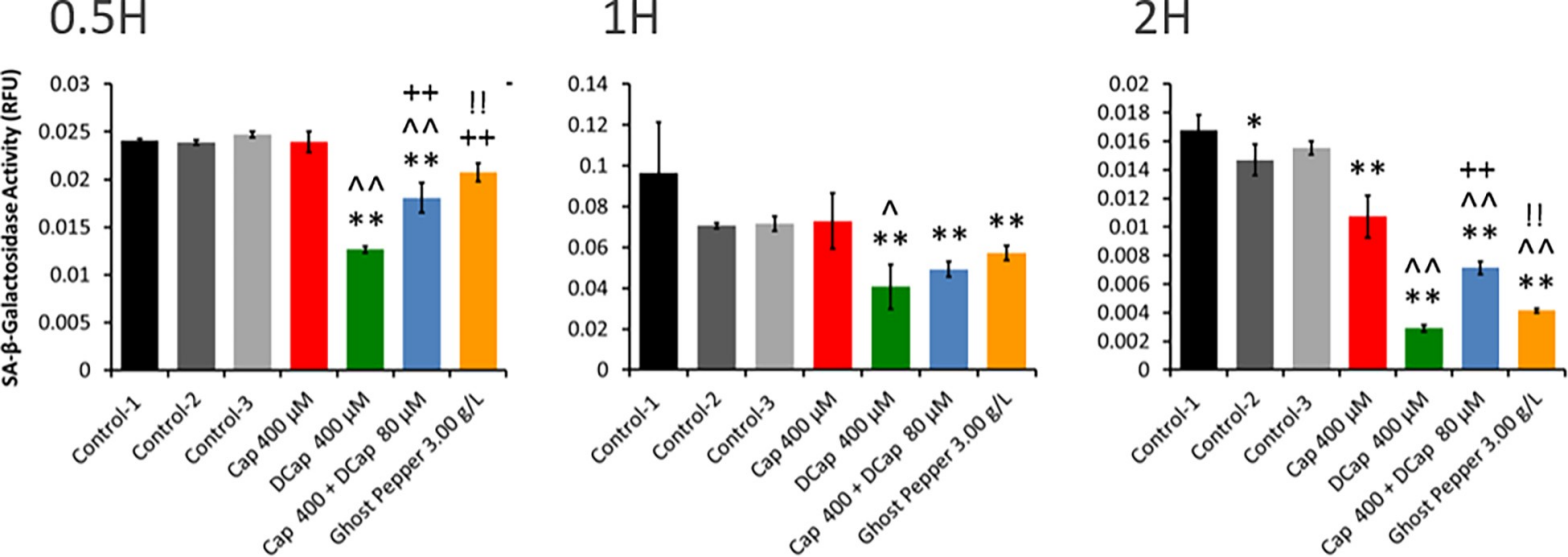
SA-beta-gal activity in human renal adenocarcinoma cells at 0.5, 1 and 2 H. Control-1 contained culture media without ethanol. Control-2 and control-3 contained culture media with different levels of ethanol (0.80 and 0.96%, respectively). Control-2 was a corresponding control for capsaicin (Cap 400 μM), dihydrocapsaicin (DCap 400 μM), and ghost pepper (3 g L^-1^) treatments. Control-3 was a corresponding control for capsaicin + dihydrocapsaicin treatment (Cap 400 μM + DCap 80 μM) (5:1). SA-beta-gal activities were normalized with respective relative fluorescence unit (RFU) values obtained with CyQUANT cell proliferation assay. Values are mean ± SD; n = 4; *p* ≤ 0.05 (*, ^, + or ! ); *p* ≤ 0.01 (**, ^^, ++ or !!); * or ** as compared to control-1; ^ or ^^ as compared to Cap 400 μM; + or ++ as compared to DCap 400 μM; ! or !! as compared to Cap 400 μM + DCap 80 μM.

As compared with control(s), all treatments increased polycaspase activity in adenocarcinoma cells at 0.5H (Fig 4). This trend was continued up to 2H. At 4H, as compared to control, all treatments except dihydrocapsaicin (400 μM) maintained high polycaspase activity. Peak polycaspase activity occurred with both dihydrocapsaicin (400 μM) and capsaicin (400 μM) + dihydrocapsaicin (80 μM) at 0.5H. As compared to control-1, peak polycaspase activity occurred with Ghost pepper (3 g L^-1^) and capsaicin (400 μM) at 1 and 2H, respectively. Polycaspase activity rapidly decreased with dihydrocapsaicin followed by Ghost pepper at 4H. Overall, polycaspase activities began to decrease at 4 H. Rapid, early, and late polycaspase response was noticed with dihydrocapsaicin, Ghost pepper and capsaicin, respectively. SA-beta-gal activity with capsaicin (400 μM) treatment was similar to control(s) activity at 0.5 and 1H and thereafter started to decline (Fig 5). Interestingly, the activity was significantly low in other treatments from 0.5 to 2H. Overall, SA-beta-gal activity started to decrease from 2H with all treatments. Dihydrocapsaicin (400μM) produced the lowest SA-beta-gal activity among all treatments at all treatment times.

### Shotgun proteomics revealed a complex network of proteins involved in Ghost pepper-treated cells

By shotgun proteomic approach, we have identified over 10,000 protein groups in each, for about 20,000 protein groups across the control and ghost pepper treated samples which map to about 68,220 protein isoforms at 6H. All the identified proteins exhibited up to several fold difference between treatment and control. Among them, approximately 8.2% (5,577) protein isoforms in Ghost pepper treated cells were significantly different from control. A selective list of proteins that were significantly affected by ghost pepper treatment are presented in Table 1 with experimental evidence. Some of the identified proteins are directly or indirectly related to apoptotic (extrinsic and intrinsic), Ras, Rb/E2F, p53, TGF-beta, WNT-beta catenin, and calcium induced cell death pathways. Several other proteins involved in methylation, acetylation, genome stability, cell cycle check point regulation, carbohydrate, protein and other metabolism and cellular mechanisms were also affected by ghost pepper treatment. Due to space constraints, key proteins and their roles in ghost pepper induced cell death are elaborated under discussion section. In summary, shotgun proteomic analysis revealed that each pathway or cellular mechanism that was affected by ghost pepper had selective upregulated as well as down regulated proteins in human renal cell adenocarcinoma at 6 H (see data in Dryad, doi:10.5061/dryad.d0s2gm0).

**Table 1:** Selective list of proteins that were affected by ghost pepper (3 g L^-1^) at 6 H.

S. No.	Protein	Gene name	Description	Protein Intensity Values*				Fold change^$^ (C/T)	*p* value^	Experimental evidence^#^
				Control (C)		Ghost Pepper (T)				
				Mean	SD	Mean	SD			
1	A0A024R374	*CTSB*	Cathepsin B, isoform CRA_a	385.36	20.01	210.87	77.26	1.83	0.038	UR, protein inferred from homology
2	C9JL25	*HSPD1*	60 kDa heat shock protein, mitochondrial	611.49	212.73	1589.49	348.72	-2.60	0.036	
3	H0YF14	*BCLAF1*	Bcl-2-associated transcription factor 1	8.59	13.14	38.67	19.92	-4.50	0.028	UR, protein predicted
4	I3L276	*HMOX2*	Heme oxygenase 2	64.98	110.82	208.33	144.13	-3.21	0.031	UR, protein
5	O43715	*TRIAP1*	TP53-regulated inhibitor of apoptosis 1	49.04	19.75	1.00	0.00	49.04	0.052	Protein
6	P04040	*CAT*	Catalase	37.92	55.59	236.27	118.47	-6.23	0.043	Protein
7	P06681	*C2*	Complement C2	1.00	0.00	3.14	0.09	-3.14	0.001	Protein
8	P19440	*GGT1*	Gamma-glutamyltranspeptidase 1	106.01	40.01	191.53	16.71	-1.81	0.032	Protein
9	P51398	*DAP3*	28S ribosomal protein S29, mitochondrial	73.78	59.58	277.07	55.12	-3.76	0.001	Protein
10	P60602	*ROMO1*	Reactive oxygen species modulator 1	1.75	1.29	41.46	2.47	-23.74	<0.001	Protein
11	P99999	*CYCS*	Cytochrome c	127.48	29.51	26.14	21.19	4.88	0.041	Protein
12	Q12888	*TP53BP1*	Tumor suppressor p53-binding protein 1	65.68	64.28	342.75	76.21	-5.22	0.036	Protein
13	Q12933	*TRAF2*	TNF receptor-associated factor 2	18.41	15.87	43.61	15.48	-2.37	<0.001	Protein
14	Q4LDX3	*JAK1*	Janus kinase 1	56.37	22.82	242.90	33.34	-4.31	0.029	UR, transcript
15	Q5TCM1	*SOD2*	Superoxide dismutase [Mn], mitochondrial	85.17	35.32	533.48	75.31	-6.26	0.003	Protein
16	Q6LCB0	*FADD*	FADD protein	46.88	19.46	4.60	6.24	10.19	0.049	UR, protein predicted
17	Q8NC60	*NOA1*	Nitric oxide-associated protein 1	12.51	12.39	79.27	29.34	-6.33	0.022	Protein
18	Q96FV9	*THOC1*	THO complex subunit 1	25.48	21.26	108.04	20.66	-4.24	0.001	Protein
19	Q96G23	*CERS2*	Ceramide synthase 2	7.61	11.45	58.99	20.92	-7.75	0.013	Protein
20	Q96NN9	*AIFM3*	Apoptosis-inducing factor 3	28.45	6.83	2.60	2.78	10.92	0.017	Protein
21	Q99439	*CNN2*	Calponin-2	883.51	220.67	632.19	165.33	1.40	0.036	Protein
22	Q9HBQ7	*CTSL*	Cathepsin L, isoform CRA_b	119.88	41.31	5.83	8.36	20.57	0.031	UR, transcript
23	Q9NP84	*TNFRSF12A*	Tumor necrosis factor receptor superfamily member 12A	1.00	0.00	50.27	6.83	-50.27	0.006	Protein
24	Q9UC56	*HSPA9*	Stress-70 protein, mitochondrial	3109.41	588.93	5240.18	193.78	-1.69	0.037	Protein
25	R4GNE4	*GPX4*	Glutathione peroxidase	27.68	24.48	143.12	33.62	-5.17	0.032	UR, protein
26	W8Q444	*SOD-1*	Superoxide dismutase-1	10.31	8.55	34.38	0.73	-3.34	0.039	UR, protein predicted
27	B0LPH5	*PRKCA*	Protein kinase C, alpha	1.00	0.00	32.06	6.67	-32.06	0.015	UR, protein predicted
28	B2R841		Serine/threonine-protein kinase PLK	25.18	20.07	114.95	18.14	-4.56	<0.001	UR, transcript
29	H3BLV9	*SRPK1*	SRSF protein kinase 1	51.13	20.64	172.98	25.24	-3.38	<0.001	UR, protein
30	P41240	*CSK*	Tyrosine-protein kinase CSK	29.24	17.70	57.75	24.52	-1.97	0.036	Protein
31	P49137	*MAPKAPK2*	MAP kinase-activated protein kinase 2	4.44	5.96	25.34	6.36	-5.71	0.028	Protein
32	Q9HC98	*NEK6*	Serine/threonine-protein kinase Nek6	4.34	2.96	12.88	3.22	-2.97	0.005	Protein
33	X5DP03	*STK39*	Serine threonine kinase 39 isoform B	12.95	10.70	109.18	33.11	-8.43	0.019	UR, transcript
34	F5H6R7	*RAP1B*	Ras-related protein Rap-1b	64.63	12.98	158.16	15.86	-2.45	0.002	UR, protein
35	F8VUA5	*RAB5B*	Ras-related protein Rab-5B	1.00	0.00	36.29	11.54	-36.29	0.034	UR, protein
36	H0YEI0	*RASSF7*	Ras association domain-containing protein 7	4.57	6.18	51.57	6.22	-11.29	<0.001	UR, protein predicted
37	Q53EX5		RAB7, member RAS oncogene family-like 1 variant	1.00	0.00	50.66	17.75	-50.66	0.040	UR, transcript
38	Q86WH2	*RASSF3*	Ras association domain-containing protein 3	1.00	0.00	3.04	0.05	-3.04	<0.001	Protein
39	B5BU32	*TK1*	Thymidine kinase	33.53	13.44	109.04	20.27	-3.25	0.048	UR, transcript
40	Q15291	*RBBP5*	Retinoblastoma-binding protein 5	5.13	4.10	83.20	22.88	-16.22	0.034	Protein
41	Q14999	*CUL7*	Cullin-7	1.62	1.08	4.93	1.63	-3.03	0.044	Protein
42	Q5VUP6	*CCAR1*	Cell division cycle and apoptosis regulator protein 1	36.96	32.65	288.28	56.87	-7.80	0.013	Protein
43	Q9UE85	*PML*	Promyelocytic leukemia gene protein	2.20	2.08	91.49	36.45	-41.58	0.046	UR, protein predicted
44	O00632	*MEN1*	Menin	1.64	1.11	44.76	16.25	-27.24	0.043	Protein
45	O43219	*TGFBI*	Transforming growth factor-beta-induced protein ig-h3	2.53	2.65	102.06	32.07	-40.37	0.028	Protein
46	Q9NYJ8	*TAB2*	TGF-beta-activated kinase 1 and MAP3K7-binding protein 2	1.00	0.00	5.75	0.06	-5.75	<0.001	Protein
47	B4DGU4	*CTNNB1*	Catenin beta-1	232.42	60.04	383.84	50.49	-1.65	0.002	UR, protein
48	P35221	*CTNNA1*	Catenin alpha-1	1239.53	50.27	1050.70	87.86	1.18	0.025	Protein
49	Q9NQB3	*TCF7L2*	Transcription factor 7-like 2	1.00	0.00	3.06	0.02	-3.06	<0.001	Protein
50	G3V361	*CALM1*	Calmodulin	735.69	97.72	297.04	49.00	2.48	0.012	UR, protein
51	H0Y9S7	*ATP2C1*	Calcium-transporting ATPase type 2C member 1	4.38	5.85	82.00	12.83	-18.72	0.012	UR, protein
52	Q13526	*PIN1*	Peptidyl-prolyl cis-trans isomerase NIMA-interacting 1	101.40	34.62	12.72	6.11	7.97	0.033	Protein
53	Q8IWX8	*CHERP*	Calcium homeostasis endoplasmic reticulum protein	45.89	13.40	228.61	7.60	-4.98	0.002	Protein
54	Q8NE86	*MCU*	Calcium uniporter protein, mitochondrial	51.21	48.90	222.46	38.65	-4.34	0.021	Protein
55	Q9H712	*CARF*	Calcium-responsive transcription factor	1.69	1.19	3.85	1.71	-2.28	0.019	Protein
56	Q9ULQ1	*TPCN1*	Two pore calcium channel protein 1	1.00	0.00	27.70	1.27	-27.70	0.001	Protein
57	Q9Y6Y1	*CAMTA1*	Calmodulin-binding transcription activator 1	7.99	3.45	4.43	4.03	1.81	0.049	Protein
58	Q16566	*CAMK4*	Calcium/calmodulin-dependent protein kinase type IV	1.00	0.00	2.91	0.21	-2.91	0.004	Protein
59	A6N6J7	*JARID1C*	JARID1C protein	8.63	13.22	24.33	11.86	-2.82	0.031	UR, transcript
60	F8WAX2	*PCMT1*	Protein-L-isoaspartate(D-aspartate) O-methyltransferase	21.35	4.97	1.00	0.00	21.35	0.019	
61	H7C2I1	*PRMT1*	Protein arginine N-methyltransferase 1	415.06	80.67	836.76	116.95	-2.02	0.020	UR, protein
62	K7EMU8	*DNMT1*	DNA (cytosine-5)-methyltransferase 1	6.38	9.31	28.80	9.81	-4.52	0.001	UR, protein
63	P22087	*FBL*	rRNA 2'-O-methyltransferase fibrillarin	472.15	13.49	910.54	78.17	-1.93	0.007	Protein
64	Q15047	*SETDB1*	Histone-lysine N-methyltransferase SETDB1	2.45	2.51	27.03	2.34	-11.03	0.006	Protein
65	Q672J1	*WHSC1*	Histone-lysine N-methyltransferase NSD2	16.83	25.92	93.37	51.46	-5.55	0.038	Protein
66	Q96KB0	*MINA*	Bifunctional lysine-specific demethylase and histidyl-hydroxylase MINA	8.62	11.57	115.01	35.32	-13.34	0.045	Protein
67	Q9HBJ3	*KMT2A*	Histone-lysine N-methyltransferase 2A	19.02	6.52	6.08	5.04	3.13	0.004	Protein
68	Q9UI30	*TRMT112*	Multifunctional methyltransferase subunit TRM112-like protein	249.20	8.85	131.01	21.43	1.90	0.016	Protein
69	Q9Y483	*MTF2*	Metal-response element-binding transcription factor 2	3.73	3.31	21.39	3.64	-5.73	0.016	Protein
70	A1Z5I6	*MDC1*	Mediator of DNA damage checkpoint 1 variant 2	1.00	0.00	14.60	3.40	-14.60	0.020	UR, transcript
71	B4DTU4	*LIG1*	DNA ligase	59.25	13.36	364.20	85.60	-6.15	0.019	UR, protein
72	B7ZVY7	*CDC2L1*	Cell division cycle 2-like 1 (PITSLRE proteins)	4.10	5.36	39.94	13.83	-9.75	0.049	UR, protein
73	H0Y9J8	*RAD17*	Cell cycle checkpoint protein RAD17	1.00	0.00	6.01	0.10	-6.01	<0.001	UR, protein
74	Q13042	*CDC16*	Cell division cycle protein 16 homolog	10.88	7.34	38.82	12.02	-3.57	0.040	Protein
75	Q15819	*UBE2V2*	Ubiquitin-conjugating enzyme E2 variant 2	352.73	88.39	3.05	2.23	115.67	0.020	Protein
76	Q16695	*HIST3H3*	Histone H3.1t	332.58	28.97	526.29	72.26	-1.58	0.018	Protein
77	Q7Z2J1	*ATRX*	Transcriptional regulator ATRX	10.14	9.15	127.09	24.60	-12.53	0.006	Protein
78	Q8WWL7	*CCNB3*	G2/mitotic-specific cyclin-B3	11.73	5.62	32.65	2.58	-2.78	0.013	Protein
79	Q9H9Q9	*CCAR2*	Cell cycle and apoptosis regulator protein 2	365.06	34.83	609.51	58.77	-1.67	0.035	Protein
80	Q9NYB0	*TERF2IP*	Telomeric repeat-binding factor 2-interacting protein 1	19.33	13.11	77.75	13.83	-4.02	0.029	Protein
81	Q9UJX4	*ANAPC5*	Anaphase-promoting complex subunit 5	18.67	17.87	128.20	22.18	-6.87	0.011	Protein
82	B4DHB3		Phosphoglycerate kinase	1176.88	352.46	362.35	135.41	3.25	0.028	UR, transcript
83	B4DPM0		Pyruvate kinase	283.82	35.20	78.39	7.42	3.62	0.014	UR, transcript
84	B4E2U0		6-phosphogluconate dehydrogenase, decarboxylating	290.03	65.21	123.93	54.62	2.34	0.002	UR, transcript
85	E9PK47	*PYGL*	Alpha-1,4 glucan phosphorylase	107.81	11.36	34.67	6.25	3.11	0.015	UR, protein
86	F2Z393	*TALDO1*	Transaldolase	593.53	58.68	288.03	71.42	2.06	0.034	UR, protein
87	H3BR68	*ALDOA*	Fructose-bisphosphate aldolase A	624.56	31.44	181.07	28.45	3.45	0.001	UR, protein
88	H3BU13	*PKM*	Pyruvate kinase PKM	943.35	81.53	566.89	16.86	1.66	0.014	UR, protein
89	H3BUH7	*ALDOA*	Fructose-bisphosphate aldolase A	2238.98	467.51	522.35	48.65	4.29	0.028	UR, protein
90	K7EM90	*ENO1*	Enolase	2210.71	635.82	1102.74	269.53	2.00	0.036	UR, protein
91	K7ENA0	*GPI*	Glucose-6-phosphate isomerase	368.63	66.05	85.26	75.23	4.32	<0.001	UR, protein
92	P09467	*FBP1*	Fructose-1,6-bisphosphatase 1	7.76	6.05	12.31	6.50	-1.59	0.020	Protein
93	P52209	*PGD*	6-phosphogluconate dehydrogenase, decarboxylating	377.41	54.26	187.45	41.03	2.01	0.010	Protein
94	Q6FI37	*IDH1*	Isocitrate dehydrogenase [NADP]	313.47	98.23	42.60	5.33	7.36	0.045	UR, transcript
95	Q9UF08	*AGL*	Glycogen debranching enzyme	29.74	13.50	59.31	19.42	-1.99	0.018	Protein
96	U3KPS5	*TPI1*	Triosephosphate isomerase	377.22	28.00	199.33	46.94	1.89	0.012	UR, protein
97	U3KQP4	*ENO2*	Gamma-enolase	56.15	22.11	8.23	12.52	6.83	0.018	UR, protein
98	F8VS27	*TSFM*	Elongation factor Ts, mitochondrial	1.00	0.00	55.29	6.52	-55.29	0.005	UR, protein
99	P39023	*RPL3*	60S ribosomal protein L3	647.05	227.22	1161.78	211.18	-1.80	0.001	Protein
100	Q05639	*EEF1A2*	Elongation factor 1-alpha 2	3113.93	773.57	1862.76	488.17	1.67	0.017	Protein
101	Q6PI48	*DARS2*	Aspartate—tRNA ligase, mitochondrial	138.13	13.89	317.58	15.14	-2.30	<0.001	Protein
102	Q9Y3D9	*MRPS23*	28S ribosomal protein S23, mitochondrial	8.12	12.33	58.22	8.90	-7.17	0.003	Protein
103	D6RHI2	*ELOVL6*	Elongation of very long chain fatty acids protein	1.00	0.00	5.72	0.01	-5.72	<0.001	UR, protein inferred from homology
104	F8VQZ7	*METAP2*	Methionine aminopeptidase 2	139.59	53.31	6.09	8.81	22.93	0.044	UR, protein
105	P51648	*ALDH3A2*	Fatty aldehyde dehydrogenase	39.06	25.75	234.48	18.06	-6.00	0.012	Protein
106	Q02880	*TOP2B*	DNA topoisomerase 2-beta	77.70	7.32	272.66	6.13	-3.51	0.001	Protein
107		Q6ICD2	*EIF3L*	Eukaryotic translation initiation factor 3 subunit L	419.15	75.34	220.10	68.41	1.90	0.002		Protein

*Area under the curve of peptide peak (no units).

^$^Fold change of a protein is a ratio of its expression in control and ghost pepper treated cells (n = 3). Negative (-) or positive (none) sign under 'fold change' column indicates over or under expression of that protein, respectively in ghost pepper treated cells as compared to control.

^Paired Student's t-test with two-tailed distribution.

^#^Experimental evidence were obtained from Swiss-Prot website. UR = Unreviewed (protein sequence and functional information are not yet reviewed at Swiss-Prot website).

## Discussion

In this study, we have examined the effect of ghost pepper (Bhut Jolokia), one of the hottest chili peppers in the world, on cell proliferation, apoptosis, senescence and the global proteomic profile in human renal cell adenocarcinoma in vitro. Results of these findings are discussed here in detail.

### Ghost pepper and major capsaicinoids had similar effects on adenocarcinoma cell proliferation

Our HPLC analysis of commercial Ghost pepper powder revealed a ratio of 5:1 for major capsaicinoids in terms of capsaicin and dihydrocapsaicin (Fig 1). In general, the ratio of capsaicin to dihydrocapsaicin ranges from 1:1 to 2:1 in chilies [6] depending on the pepper source and method of extraction [10]. All our experiments were designed to reflect the ratio of capsaicin to dihydrocapsaicin in the purchased ghost pepper powder. The ratio of major capsaicinoids in the ethanol extracts of ghost pepper may not be exactly equal to the ratio estimated by HPLC. However, this ratio serves as a reference for cell culture experiments.

Various concentrations of capsaicin, dihydrocapsaicin, capsaicin + dihydrocapsaicin and ghost pepper had the similar effect on adenocarcinoma cell proliferation (Fig 2). Therefore, ghost pepper could exert its effects on cell proliferation via capsaicin and dihydrocapsaicin. However, the role of other minor capsaicinoids of ghost pepper in anticancer properties cannot be ignored. The anticancer effects of capsaicin in vitro were previously found to be both dose- and time-dependent [23]. Bley et al. [12] found that the effects of capsaicin are in the low micromolar range and become maximal at approximately 200 to 300μM. The duration of exposure enhances the potency of capsaicin and its stability under specific experimental conditions. Recent studies have confirmed and extended these observations to additional cell lines and to rodent in vivo xenograft tumor models [12]. Together, capsaicin and dihydrocapsaicin at 5:1 ratio did not show any additive or synergistic effects on cell proliferation compared to either compound alone. We are not sure of specific reasons for this kind of response in human renal adenocarcinoma cells. Data pertaining to cell proliferation in various controls indicate that ethanol, the solvent we used to dissolve capsaicinoids, did not significantly affect the proliferation of human adenocarcinoma cells in vitro (Fig 3).

### Apoptosis versus senescence

Ghost pepper and its major capsaicinoids induced early polycaspase responses in human renal adenocarcinoma cells in vitro (Fig 4). The polycaspase FLICA probe, FAM-VAD-FMK, detects apoptosis by recognizing different types of activated caspases in a cell. On the other hand, SA-beta-gal is considered one of the markers of senescence in cells [24]. In this study, SA-beta-gal activity was normal or was downregulated with various treatments in human renal adenocarcinoma cells (Fig 5). The mechanism that directs whether a cell undergo apoptosis or senescence is unknown. These two processes seem to be exclusive [25]. Our results indicate that ghost pepper, capsaicin and dihydrocapsaicin induce apoptosis rather than senescence in human renal cell adenocarcinoma in vitro. Thus, the antiproliferative activity of the treatments we tested could be attributed to their ability to induce apoptosis with various caspases. Capsaicin appears to induce apoptosis in more than 40 different cancer cell lines, mostly human cancer lines [12]. The mechanisms of capsaicin-induced apoptosis have been discussed in detail [1,12]. One or more of these mechanisms are probably responsible for the apoptosis we found. These mechanisms include inhibition of mitochondrial respiration; suppression of plasma membrane NADH-oxidoreductase; significant elevation of intracellular reactive oxygen species production; blocking cell cycle progression and triggering apoptosis by downregulating cyclin D1; and suppression of activation, nuclear translocation and/or DNA binding of STAT [1]. Other mechanisms proposed to play a role in anticancer activities of capsaicin include antioxidant activity, activation of peroxisome proliferator-activated receptor gamma, inhibition of angiogenesis, modulation of lipid metabolism, and/or inhibition of aromatase activity [12]. The mechanisms associated with anticancer activities of capsaicin are complex. Normal or noncancerous cells appear to be significantly less sensitive to the apoptotic or growth inhibitory effects of capsaicin than cancerous cells [12]. Earlier video microscopy studies revealed that dynamic morphologic changes in cell culture take place in less than 2h. An estimated duration of an apoptotic cell death is in between 6 and 24h in vivo. This duration is influenced by type of cells undergoing apoptosis [26].

### Ghost pepper modulates a complex network of proteins

Data pertaining to global proteomic analysis further supports the results of cell proliferation and apoptosis (Table 1**)**. Data suggests that ‘fold change’ of a protein does not alone explain any difference between control and treatment. For this reason, *p* value (≤ 0.05) was considered along with fold change value to draw conclusion on a protein. Selective proteins that were significantly affected by ghost pepper are discussed below in detailed.

### Intrinsic and extrinsic apoptotic pathways

Proteins that were upregulated or down regulated by ghost pepper treatment in adenocarcinoma cells at 6 H appeared to be late-responsive and/or stably expressed proteins. In general, caspases are activated by pro-apoptotic proteins released from mitochondria which ultimately induce apoptosis [27]. In the present study, polycaspases were induced at 0.5 H, and their expression started to decrease at 4 H (Fig 4). Similarly, cytochrome C (P99999; *CYCS*) [28] and FADD proteins (Q6LCB0; *FADD*) [29], which activate different caspases, were also decreased at 6 H in Ghost pepper treated cells as compared to control (Table 1). Apoptosis-inducing factor 3 (Q96NN9, *AIFM3* or *AIFL*) when expressed heterologously induce apoptosis in a caspase-dependent manner [30]. Decreased AIFM3 levels in Ghost pepper treatment were also in harmony with caspase levels. Because of this dynamic nature, certain early-responsive proteins may or may not appear at a later time during Ghost-pepper treatment. Furthermore, visual observations under microscope at 6 H indicate that most of the cells exhibited symptoms of apoptosis in Ghost pepper treated cells (personal communications). To date, three pathways viz., extrinsic (death receptor pathway), intrinsic (mitochondrial pathway), and perforin/granzyme pathways, are established for apoptosis. All these three pathways have same terminal execution pathway which involves caspase-3, DNA fragmentation, cytoskeletal and nuclear protein degradation, protein cross linking, formation of apoptotic bodies, and removal of dead cells. The perforin/granzyme pathway of apoptosis is a non-caspase pathway involves single stranded DNA damage [31]. The intrinsic pathway is current target for tumor suppression studies [32], and it is initiated within the cell in response to DNA damage, severe cellular stress, or loss of survival factors in the cells [33]. Up regulation of reactive oxygen species modulator 1 (P60602; *ROMO1*), mitochondrial superoxide dismutase (Q5TCM1; *SOD2*), superoxide dismutase-1 (W8Q444; *SOD-1*), catalase (P04040; *CAT*), glutathione peroxidase (R4GNE4; *GPX4*), gamma-glutamyltranspeptidase 1 (P19440; *GGT1*), and heme oxygenase 2 (I3L276; *HMOX2*) in cells treated by ghost pepper indirectly suggests that these cells were under severe oxidative stress. Furthermore, over production of other stress response proteins viz., mitochondrial stress-70 protein (Q9UC56; *HSPA9*), and mitochondrial 60 kDa heat shock protein (C9JL25; *HSPD1*), also indicates cellular stress in ghost pepper treated cells. B-cell lymphoma 2 (Bcl-2) family of proteins regulate intrinsic pathway by controlling pro- and anti-apoptotic signals in the cell [33]. In the present study, Bcl-2 associated transcription factor 1 (H0YF14; *BCLAF1*) was significantly over expressed in ghost pepper treatment at 6 H. Bcl-2 associated transcription factor 1 protein is known to interact with several members of the Bcl-2 family of proteins and its overexpression induces apoptosis or cell cycle arrest [34]. Increased tumor suppressor p53-binding protein 1 (Q12888; *TP53BP1*) was probably involved in DNA-damage signaling pathways in ghost pepper treated cells [35]. As TP53-regulated inhibitor of apoptosis 1 (O43715; *TRIAP1*) was decreased in treated cells, its role in inhibiting activation of caspase-9 and prevention of apoptosis induction [36] was probably negatively affected by Ghost pepper at 6 H. However, tumor necrosis factor receptor superfamily member 12A (Q9NP84; *TNFRSF12A*) and TNF receptor-associated factor 2 (Q12933; *TRAF2*) were still expressed more in treated cells at 6 H. Both of these proteins’ roles were implicated in positive regulation of extrinsic apoptotic pathway [37]. Similarly, over expression of janus kinase 1 (Q4LDX3; *JAK1*) protein in treated cells indicates its role in ghost pepper induced early signaling events related to cytokine receptors [38]. Ceramide, a membrane sphingolipid metabolite, is synthesized de novo by ceramide synthase. It perpetuates cellular stress response and induces apoptosis, terminal differentiation, or cell cycle arrest [39,40]. Ceramide induces apoptosis by activating caspases, especially caspase 3 [41], endonucleases that are responsible for DNA cleavage [42], and by regulating release of cytochrome C [41]. It also plays a role in G0/G1 cell cycle arrest through retinoblastoma gene product [41]. Over expressed ceramide synthase 2 (Q96G23; *CERS2*) might have played a role in apoptosis and/or cell cycle arrest through ceramide production in ghost pepper treated cells in the present study. Over expressed nitric oxide-associated protein 1 (Q8NC60; *NOA1*), a large mitochondrial GTPase, might have also supported apoptosis process in treated cells by interacting with complex I of the electron transport chain, and DAP3 (death-associated protein 3), a positive regulator of apoptosis [43]. These findings are further supported by over expression of mitochondrial 28S ribosomal protein S29 (P51398), another name for DAP3, in Ghost pepper treated cells. Furthermore, increased levels of THO complex subunit 1 (Q96FV9 or p84N5; *THOC1*) in ghost pepper treatment suggest that apoptotic pathway different from those activated by death domain-containing receptors or p53 was active in the treated cells [44].

Down regulation of calponin-2 (Q99439; *CNN2*) in Ghost pepper treated cells might be associated with morphological changes and detachment of cancer cells [45]. Furthermore, increased levels of the complement C2 of the innate immunity (P06681; *C2*) appears to be associated with clearance of immune complexes and apoptotic materials [46]. Lysosomal cell death (LCD) is mainly carried out by the lysosomal cathepsin proteases, and their inhibition do not give full proof protection from LCD [47]. Cathepsin mediated LCD is not appears to be a major event in Ghost pepper treated cells at 6 H. This is evident from down regulation of two cathepsins viz., cathepsin L, isoform CRA_b (Q9HBQ7; *CTSL*) and cathepsin B, isoform CRA_a (A0A024R374; *CTSB*) in the treated cells.

### *Ras* pathway

Cell growth and proliferation are influenced by extracellular signals. Pathways of cell proliferation are generally initiated by activation of a receptor tyrosine kinase by a growth factor. The *Ras* pathway is considered as an important one among various cell proliferation pathways. Key components of the *Ras* pathway are a cascade of serine/threonine kinases, a mitogen–activated protein kinase (MAPK), and transcription factors like FOS and JUN [48]. In the present study, many kinases (tyrosine-protein kinase CSK, P41240, *CSK*; serine/threonine-protein kinase PLK, B2R841; serine/threonine-protein kinase Nek6, Q9HC98, *NEK6*; serine threonine kinase 39 isoform B, X5DP03, *STK39*; SRSF protein kinase 1, H3BLV9, *SRPK1*; calcium/calmodulin-dependent protein kinase type IV, Q16566, *CAMK4*; protein kinase C alpha, B0LPH5, *PRKCA*; and MAP kinase-activated protein kinase 2, P49137, *MAPKAPK2*) were over expressed in Ghost pepper treatment (Table 1). A similar trend was observed in *Ras* specific proteins (RAB7, member RAS oncogene family-like 1 variant, Q53EX5; *Ras* association domain-containing protein 3, Q86WH2, *RASSF3*; *Ras* association domain-containing protein 7, H0YEI0, *RASSF7*; Ras-related protein Rab-5B, F8VUA5, *RAB5B*; and Ras-related protein Rap-1b, F5H6R7, *RAP1B*) in ghost pepper treatment. These finding suggest that the machinery for cell proliferation was very much active while adenocarcinoma cells were undergoing apoptosis in ghost pepper treatment. However, cell proliferation data (Fig 2) confirms the dominant role of apoptosis over cell proliferation.

### Rb/E2F pathway

Retinoblastoma (Rb) and p53 family of tumor suppressor genes are considered as some of the important targets for the treatment of drug resistant-cancer patients [49]. Control of Rb/E2F pathway, which regulates initiation of DNA replication, is disrupted in virtually all human cancers. Thymidine kinase which is involved in nucleotide biosynthesis during initiation of DNA replication is under the control of E2F [50]. Increased thymidine kinase (B5BU32; *TK1*) level is an indication of disrupted Rb/E2F pathway and ongoing cell cycle activity in the dying Ghost pepper treated cells (Table 1). Retinoblastoma-binding protein 5 (Q15291; *RBBP5*) is another Rb related protein overexpressed in the treated cells. It is an important component required for the activity of methyltransferases involved in histone H3 Lys-4 methylation [51].

### P53 pathway

Tumor suppressor p53 protein is a transcriptional regulator. It activates expression of several genes involved in cell death, cell cycle arrest, senescence and DNA-repair. Perhaps, it is inactivated in most cancers [52]. Over expressed ‘promyelocytic leukemia gene protein’ (Q9UE85; *PML*) might be an essential component of Ghost pepper induced stress or DNA damage-activated apoptotic pathways (Table 1). PML is known for its role in the pathogenesis of acute promyelocytic leukemia (APL). It is not only involved p53-dependent apoptosis but also in FAS and TNFα-induced apoptosis [53]. Furthermore, cytoplasmic PML is considered as a critical regulator of TGF-beta. Often, TGF-beta signaling is deregulated in cancer [54]. Over expression of ‘cell division cycle and apoptosis regulator protein 1’ (Q5VUP6; *CCAR1* or *CARP-1*) might have a role in regulation of expression of key cell proliferation-inducing genes, and might have acted as a p53 coactivator. Expression of CARP-1 induces apoptosis but affected by expression of c-Myc or 14-3-3 [55]. Increased levels of cullin7 (Q14999; *CUL7*), a novel oncogene that promote cell proliferation and invasion by suppressing p53 expression [56], indicates its role against p53-dependent apoptosis ghost pepper treated cells.

### TGF-Beta pathway

Transforming growth factor-beta (TGF-beta) signalling has either a tumour-suppressing or tumour-promoting function depending on cellular context [57]. A few TGF-beta pathway associated proteins were elevated in ghost pepper treatment. Among them, ‘TGF-beta-activated kinase 1 and MAP3K7-binding protein 2’ (Q9NYJ8; *TAB2*) is a novel adaptor protein known to stimulate TAK1 MAPKKK by linking TAK1 to TRAF6 in the IL-1 signal transduction pathway during the inflammation process (Table 1) [58]. Another TGF-beta pathway related protein was TGF-beta-induced protein ig-h3 (O43219; *TGFBI*). TGFBI silencing effectively reduces cell proliferation and elevates motility of melanoma cells in vitro [59]. Whereas, in mesothelioma and breast cancer cells, TGFBI suppress cell proliferation, delay G1-S phase transition, and induces death [60]. Another protein that influences TGF-beta pathway is menin (O00632; *MEN1*). Its suppression antagonizes TGF-beta mediated cell growth inhibition [61]. Its over expression probably having opposite role in ghost pepper treatment.

### WNT-beta catenin pathway

In general, wnt signaling pathways offer less specificity for candidate drugs. These pathways include the Wnt/beta-catenin pathway, and beta-catenin-independent pathways (the planar cell polarity (PCP) pathway and Wnt/ Ca2+ pathway) [62]. Among them, transcription factor 7-like 2 (Q9NQB3; *TCF7L2* or *TCF-4*); and catenin beta-1 (B4DGU4; *CTNNB1*) proteins were over expressed in ghost pepper treatment (Table 1). Perhaps, over expression of TCF-4 and CTNNB1 genes were associated with the activation of MAPK gene in breast cancer cell lines with different degrees of invasiveness [63]. Whereas, chondrocyte apoptosis in osteoarthritis was induced by elevated TCF-4 mRNA expression through NF-κB signaling [64]. On the other hand, a decreased expression levels of catenin alpha-1 (P35221; *CTNNA1* or Renal carcinoma antigen NY-REN-13) is commonly seen in gastric carcinoma patients [65].

### Calcium induced cell death pathways

Calcium homeostasis is associated with apoptosis and autophagy through shared molecular effectors and signal routes [66]. In support of this, several calcium related proteins viz., calcium/calmodulin-dependent protein kinase type IV (Q16566; *CAMK4*); two pore calcium channel protein 1 (Q9ULQ1; *TPCN1*); calcium homeostasis endoplasmic reticulum protein (Q8IWX8; *CHERP*); calcium-transporting ATPase type 2C member 1 (H0Y9S7; *ATP2C1*); calcium-responsive transcription factor (Q9H712; *CARF*); and mitochondrial calcium uniporter protein (Q8NE86; *MCU*) were significantly increased by Ghost pepper treatment (Table 1). Role of Q16566 (*CAMK4*) in regulation of MAP kinase pathways and related transcription [67]; Q9ULQ1 (*TPCN1 or TPC1*) in ion channel regulation through the interaction of an anti-apoptotic Hax-1 protein [68]; Q8IWX8 (*CHERP*) in cell cycle arrest [69]; Ca^2+^-ATPases (pumps), such as H0Y9S7 (*ATP2C1*), in regulation of Ca^2+^ to low levels in eukaryotic cells [70]; Q9H712 (*CARF*) in Ca^2+^ responsive transcription [71]; Q8NE86 (*MCU*) along with MICU1 in controlling Ca^2+^ uptake and related overload stress in mitochondria [72]; and calmodulin-binding transcription activator 1 (Q9Y6Y1; *CAMTA1*) in growth suppression [73] was already documented. Apart from CAMTA1, other proteins viz., calmodulin (G3V361; *CALM1*) and peptidyl-prolyl cis-trans isomerase NIMA-interacting 1 (Q13526; *PIN1*) were significantly reduced in Ghost pepper treatment at 6 H. Furthermore, PIN1’s involvement in DNA damage mediated signaling pathway [74], and CALM1’s participation in calcium-signaling (calcium/calmodulin pathway) [75] suggest that the role of these proteins in signaling in ghost pepper treated cells appears to be much earlier than 6 h. Ca^2+^ might have also influenced various protein kinases discussed earlier, proteases and endonucleases [66].

### Methylation and acetylation

Food can influence gene expression by various epigenetic mechanisms that can affect DNA methyltransferase (DNMT), histone deacetylase (HDAC), histone acetyltransferase (HAT), or noncoding RNA expression. Diet can therefore regulate cellular longevity and carcinogenesis through these epigenetic mechanisms [76]. Several methyltransferases or related proteins viz., DNA (cytosine-5)-methyltransferase 1 (K7EMU8; *DNMT1*); histone-lysine N-methyltransferase SETDB1 (Q15047; *SETDB1*); histone-lysine N-methyltransferase NSD2 (Q672J1; *WHSC1*); rRNA 2'-O-methyltransferase fibrillarin (P22087; *FBL*); metal-response element-binding transcription factor 2 (Q9Y483; *MTF2*); protein arginine N-methyltransferase 1 (H7C2I1; *PRMT1*); menin (O00632; *MEN1*); and retinoblastoma-binding protein 5 (Q15291; *RBBP5*) were significantly increased in Ghost pepper treatment (Table 1). Similarly, demethylases or related proteins viz., JARID1C protein (A6N6J7; *JARID1C*) and bifunctional lysine-specific demethylase and histidyl-hydroxylase MINA (Q96KB0; *MINA*) were significantly increased in ghost pepper treated cells. On the other hand, some other methyltransferases or related proteins viz., histone-lysine N-methyltransferase 2A (Q9HBJ3; *KMT2A*); multifunctional methyltransferase subunit TRM112-like protein (Q9UI30; *TRMT112*); protein-L-isoaspartate (D-aspartate) O-methyltransferase (F8WAX2; *PCMT1*) were significantly decreased in Ghost pepper treatment. Majority of these proteins are involved in, chromatin regulation, transcription or transcription regulation [37]. Increased levels of Menin might have also directly repressed human telomere reverse transcriptase (hTERT), which is known to contribute to tumorigenesis [77] in ghost pepper treated cells.

### Genome stability and cell cycle check point regulation

Telomere maintenance is active in many human cancers and in vitro immortalized cell lines by a telomerase-independent pathway called as the Alternative Lengthening of Telomeres (ALT) pathway. Loss of transcriptional regulator ATRX protein (Q7Z2J1; *ATRX*) and mutations in its gene are the hallmarks of ALT-immortalized cell lines [78]. Gain of ALTRX protein in ghost pepper treated cells is an indication of negative effect of Ghost pepper on immortal behavior of adenocarcinoma cells (Table 1). However, data on SA-beta-gal suggests that these changes might not be sufficient to induce senescence in Ghost pepper treated human renal adenocarcinoma cells (Fig 5). However, increased levels of Menin (O00632), a protein involved in histone methylation, suggests that the activity of hTERT and related telomere maintenance might have suppressed in Ghost pepper treated cells [77]. On the other hand, over expressed telomeric repeat-binding factor 2-interacting protein 1 (Q9NYB0; *TERF2IP* or *TRF2*) and histone H3.1t (Q16695; *HIST3H3*) might have protected telomeres [37,79] to some extent in ghost pepper treated cells. These findings also suggest that there was a competition between maintenance and suppression of maintenance of telomeres in ghost pepper treated cells.

Cell cycle checkpoint protein RAD17 (H0Y9J8; *RAD17*) was increased in Ghost pepper treatment (Table 1). Increased levels of RAD17 are linked with breast [80] and lung carcinomas [81]. Cell cycle check point activation in response to DNA damage engage RAD9-RAD1-HUS1 complex (9-1-1) at DNA damage sites by RAD17-RFC (replication factor C) complex [82]. Increased levels of cell division cycle 2-like 1 (PITSLRE proteins; B7ZVY7; *CDC2L1*) might be involved in phosphorylation and activation of downstream factors of death execution [83]. Several other cell cycle regulating proteins were also elevated by ghost pepper treatment. Among them, the role of cell division cycle and apoptosis regulator protein 1 (Q5VUP6; *CCAR1*) in apoptosis signaling [84]; G2/mitotic-specific cyclin-B3 (Q8WWL7; *CCNB3*) in blocking the mitotic cell cycle [85]; mediator of DNA damage checkpoint 1 variant 2 (A1Z5I6; *MDC1*) in cellular response to DNA double-strand breaks [86]; cell cycle and apoptosis regulator protein (Q9H9Q9; *CCAR2* or *DBC1*) in p53-mediated apoptosis through specific inhibition of SIRT1, an NAD-dependent deacetylase [87]; anaphase-promoting complex subunit 5 (Q9UJX4; *ANAPC5* or *APC5*) in controlling cell cycle through E2F1 ubiquitination [88]; cell division cycle protein 16 homolog (Q13042; *CDC16* or *APC6*) in maintaining integrity of APC, an essential cell-cycle regulator [89]; and DNA ligase (B4DTU4; *LIG1*) in DNA repair [90] were already established. Furthermore, DNA repairing protein ubiquitin-conjugating enzyme E2 variant 2 (Q15819; *UBE2V2* or *MMS2*) [91] was heavily knocked down in ghost pepper treatment.

### Carbohydrate metabolism

Metabolic oncology is relatively a new field in cancer research and therapy [92]. Whether it is a normal or cancer cell, enough metabolic resources are required for replicative cell division to build mass of new cells. Glucose regulates cell cycle checkpoint occurs at G1/S boundary. This cell cycle inhibition occurs through phosphorylation of p53 by an intrinsic cell-regulator of the cell cycle, AMP-activated protein kinase (AMPK). AMPK synchronizes cell cycle with carbon source availability [93]. In other words, diminished glucose or carbohydrate metabolism is an indicator of dwindling proliferation of cells. In agreement with this, the levels of many proteins involved in carbohydrate metabolism viz., 6-phosphogluconate dehydrogenase, decarboxylating (B4E2U0); 6-phosphogluconate dehydrogenase-decarboxylating (P52209, *PGD*); triose phosphate isomerase (U3KPS5; *TPI1*); alpha-1,4 glucan phosphorylase (E9PK47; *PYGL*); transaldolase (F2Z393; *TALDO1*); isocitrate dehydrogenase [NADP] (Q6FI37; *IDH1*); glucose-6-phosphate isomerase (K7ENA0; *GPI*); fructose-bisphosphate aldolase A (H3BR68; *ALDOA*); pyruvate kinase (B4DPM0); pyruvate kinase PKM (H3BU13; *PKM*); gamma-enolase (U3KQP4; *ENO2*); phosphoglycerate kinase (B4DHB3); fructose-bisphosphate aldolase A (H3BUH7; *ALDOA*); and enolase (K7EM90; *ENO1*) were down regulated by Ghost pepper treatment (Table 1). Suppressed mitochondrial glucose oxidation with simultaneously enhanced cytoplasmic glycolysis inhibits apoptosis, and provides proliferative advantage to cancer cells. This led to the development of new drugs for cancer treatment. These drugs target metabolic enzymes such as pyruvate kinase, pyruvate dehydrogenase kinase, isocitrate dehydrogenase, and lactate dehydrogenase [92]. Ghost pepper reduced the levels of pyruvate kinase (B4DPM0), pyruvate kinase PKM (H3BU13; *PKM*), and isocitrate dehydrogenase [NADP] (Q6FI37; *IDH1*). Furthermore, increased levels of fructose-1,6-bisphosphatase 1 (P09467; *FBP1*), an enzyme reverse a glycolysis reaction catalyzed by phosphofructokinase [94][94]; and simultaneously decreased levels glycolysis proteins viz., glucose-6-phosphate isomerase (K7ENA0; *GPI*); fructose-bisphosphate aldolase A (H3BR68; *ALDOA*); pyruvate kinase (B4DPM0); pyruvate kinase PKM (H3BU13; *PKM*); gamma-enolase (U3KQP4; *ENO2*); phosphoglycerate kinase (B4DHB3); fructose-bisphosphate aldolase A (H3BUH7; *ALDOA*); and enolase (K7EM90; *ENO1*), indicate that cytoplasmic glycolysis was also affected in ghost pepper treatment. These findings suggest that both the mitochondrial glucose oxidation and the cytoplasmic glycolysis were affected in the ghost pepper treated cells. A decrease in the activity of the anaphase-promoting complex/cyclosome–Cdh1 (APC/C-Cdh1), an ubiquitin ligase, activates both cell proliferation and glycolysis in normal as well as neoplastic cells [95]. In the present study, increased anaphase-promoting complex subunit 5 (Q9UJX4; *ANAPC5*) was probably involved in decreased cell proliferation and glycolysis in ghost pepper treated cells. SKP1/CUL-1/F-box protein–beta-transducin repeat-containing protein (SCF-beta-TrCP) control the transient appearance and metabolic activity of the glycolysis-promoting enzyme 6-phosphofructo-2-kinase/fructose-2,6-bisphosphatase isoform 3 (PFKFB3) [96][96]. Increased levels of glycogen debranching enzyme (Q9UF08; *AGL*) in Ghost pepper treated cells appears to be an important characteristic of cancer cell pathophysiology [97].

### Protein and other metabolism

Several proteins of protein metabolism were also affected by Ghost pepper treatment. Decreased elongation factor 1-alpha 2 protein (Q05639; *EEF1A2 or Statin-S1*) (Table 1), a translation factor of protein synthesis, might have upregulated apoptosis pathway proteins (caspase3, BAD, BAX, PUMA) in ghost pepper treatment. These findings are supported by a recent study on prostate cancer tissues. In these tissues, the levels of EEF1A2 and caspase3 were inversely correlated [98]. Proteins associated with protein biosynthesis (mitochondrial aspartate-tRNA ligase, Q6PI48, *DARS2*; 60S ribosomal protein L3, P39023, *RPL3*; mitochondrial 28S ribosomal protein S23, Q9Y3D9, *MRPS23*; mitochondrial elongation factor Ts, F8VS27, *TSFM*, etc.), fatty acid metabolism (elongation of very long chain fatty acids protein, D6RHI2, *ELOVL6*; and fatty aldehyde dehydrogenase, P51648, *ALDH3A2*), DNA metabolism (DNA topoisomerase 2-beta, Q02880, *TOP2B*; thymidine kinase, B5BU32, *TK1*; and DNA ligase, B4DTU4, *LIG1*), were over expressed by ghost pepper treatment in the human kidney adenocarcinoma cells. On the other hand, some of the proteins related to protein biosynthesis (eukaryotic translation initiation factor 3 subunit L, Q6ICD2, *EIF3L*; elongation factor 1-alpha 2, Q05639, *EEF1A2*; and methionine aminopeptidase 2, F8VQZ7, *METAP2*) were under expressed by ghost pepper treatment in human kidney adenocarcinoma cells. Perhaps, various metabolic checkpoints that dictate cell fate in response to metabolic fluctuations and cell death regulation were reviewed recently [99]. Proteins involved in other metabolism and cellular mechanisms were also affected by ghost pepper (see data in Dryad, doi:10.5061/dryad.d0s2gm0). These proteins are not discussed here due to space constraints.

## Conclusions

**Fig 6:**
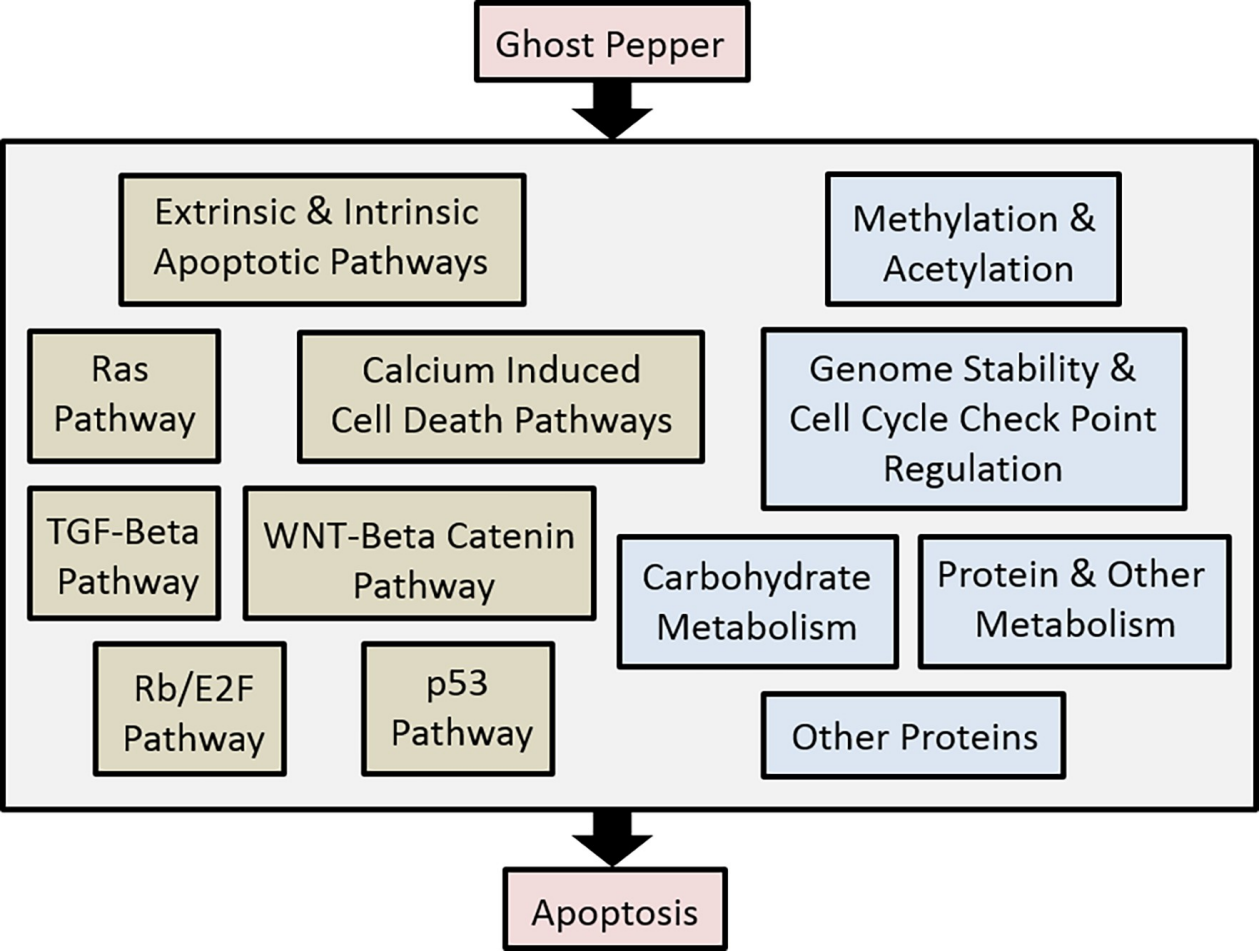
Mechanism of ghost pepper mediated cell death in human renal adenocarcinoma cells. Ghost pepper induce apoptosis by regulating the expression of key proteins involved in several cellular pathways, molecular mechanisms and metabolism. Enzymatic assays and global proteomic analysis suggest that Ghost pepper induced apoptosis in human renal adenocarcinoma cells was mediated through intrinsic and extrinsic apoptotic pathways, Ras, Rb/E2F, p53, TGF-beta, WNT-beta catenin, and calcium induced cell death pathways. Broadly two types of protein responses were noticed within each pathway. One type of proteins over expressed while other type were down regulated by ghost pepper treatment. These imbalances favored apoptosis rather than cell proliferation in ghost pepper treatment. Besides these pathways, ghost pepper also induced changes in methylation, acetylation, genome stability, cell cycle check points, carbohydrate, protein and other metabolism.

Unlike common chili peppers, ghost pepper contains very high proportions of capsaicin and dihydrocapsaicin. Dose and time dependent reduction of human adenocarcinoma cell proliferation was observed with capsaicin, dihydrocapsaicin and ghost pepper extract in vitro. Reduced cell proliferation with these treatments was ascribed to apoptosis rather than senescence. This was evident from upregulation of early polycaspase activities, and normal or suppressed senescence specific SA-beta-gal activity in the treated cells. In summary, global proteomic analysis revealed that ghost pepper induced apoptosis in human renal adenocarcinoma cells was mediated through intrinsic and extrinsic apoptotic pathways, Ras, Rb/E2F, p53, TGF-beta, WNT-beta catenin, and calcium induced cell death pathways (Fig 6). Broadly two types of protein responses were noticed within each pathway. One type of proteins over expressed while other type was downregulated by ghost pepper treatment. These imbalances probably favored apoptosis rather than cell proliferation. Besides these pathways, ghost pepper also induced changes in methylation, acetylation, genome stability, cell cycle check points, carbohydrate, protein and other metabolism. Several other proteins were also affected by ghost pepper in human renal adenocarcinoma cells (see data in Dryad, doi:10.5061/dryad.d0s2gm0). Further in depth studies are required before making any conclusion on ghost pepper for clinical applications. In future, we are planning to conduct similar experiments to understand toxic effects of ghost pepper on normal human cells including normal human kidney cells. Furthermore, time-course experiments will help us to identify a short list of candidate proteins for apoptosis in ghost pepper treatment in future.

## Abbreviations

Ex/em: Excitation/emission
H: Hours after treatment
HPLC: High-performance liquid chromatography
RFU: Relative fluorescence units
SA-beta-gal: Senescence associated-beta-galactosidase
